# Why Total Artificial Hearts Fail: Hemocompatibility, Infection, and the Biological Limits of Durable Cardiac Replacement

**DOI:** 10.7759/cureus.105988

**Published:** 2026-03-27

**Authors:** Tobias K Fuchs, Bernard Kadio

**Affiliations:** 1 Clinical Sciences, Edward Via College of Osteopathic Medicine (VCOM), Blacksburg, USA; 2 Preventive Medicine and Public Health, Edward Via College of Osteopathic Medicine (VCOM), Blacksburg, USA

**Keywords:** acquired von willebrand syndrome, biventricular failure, blood-material interaction, destination therapy, driveline infection, heart transplantation, hemocompatibility, mechanical circulatory support, neurohumoral integration, total artificial heart

## Abstract

Total artificial hearts (TAHs) can restore hemodynamics in refractory biventricular failure, yet truly durable long-term support remains difficult to achieve. This editorial examines more than five decades of TAH development, beginning with the first human implantation in 1969, and highlights recurring constraints that continue to limit performance across platforms. These include hemocompatibility problems that can drive both thrombosis and bleeding, chronic infection risk from permanent percutaneous interfaces and biofilm formation, physiologic mismatch from loss of native autoregulation and cardio-renal/endocrine signaling, immune and inflammatory activation related to foreign surfaces and recurrent infection, and persistent challenges in patient selection. Together, these patterns suggest that replacing pump output alone does not replace the heart as an integrated endocrine, immunologic, and neurohumoral organ. Recognizing these limits is essential for realistic counseling and for directing innovation toward improved blood-contacting surfaces, fully implantable energy delivery, smarter closed-loop control, and hybrid biologic-mechanical strategies.

## Editorial

Scope and approach

This editorial was conducted using targeted PubMed searches with keywords including "total artificial heart," "mechanical circulatory support," "hemocompatibility," "driveline infection," and "cardiac transplantation outcomes." Emphasis was placed on landmark device trials, registry reports, and mechanistic studies. Additional references were identified from the bibliographies of key publications. This review is not a systematic review or meta-analysis. The principal domains that limit durable total artificial heart (TAH) therapy are summarized in Table 1.

**Table 1:** Multidomain constraints limiting durable TAH therapy TAH: Total artificial heart; RV: Right ventricular; vWF: von Willebrand factor

Failure domain	Mechanism	Clinical consequence	Supporting evidence
Hemocompatibility	High shear, mechanical valves, synthetic surfaces	vWF degradation, thrombosis, bleeding	[1-8]
Driveline interface	Chronic percutaneous conduit	Biofilm infection, sepsis	[9,10]
Physiologic mismatch	Loss of endocrine and neurohumoral signaling	Impaired autoregulation	[1,11]
Immune activation	Foreign body response	Chronic inflammation, immunothrombosis	[10,12]
Patient selection	RV failure misclassification, frailty	Early mortality	[13-16]

Clinical and historical context

Evolution of TAH Development

**Figure 1:**
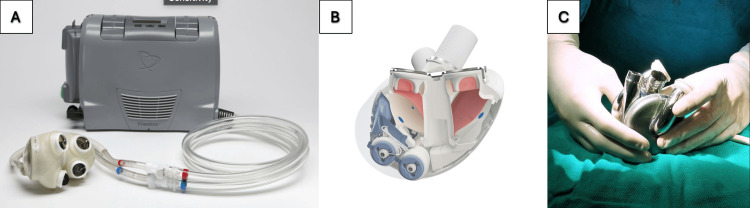
Representative TAH platforms discussed in this review A: SynCardia TAH (SynCardia Systems LLC) with external Freedom driver console; B: Aeson®  open-view device architecture; C: BiVACOR TAH intraoperative view Image credits: Panel A adapted from *SynCardia Total Artificial Heart (TAH) with Freedom Driver (Photograph)*, Wikimedia Commons, CC BY 4.0; Panel B adapted from CARMAT via EurekAlert (usage restrictions: none); Panel C adapted from BiVACOR Inc. via EurekAlert (usage restrictions: none) TAH: Total artificial heart

The first human TAH implantation was performed by Cooley on April 4, 1969, marking the beginning of complete mechanical heart replacement [11]. This pneumatic device established the paradigm of replacing native ventricles with a positive displacement pump system. Over the subsequent 50 years, TAH development has progressed through distinct technological generations: first-generation pulsatile pneumatic devices (Jarvik 7, SynCardia Systems LLC, Tucson, AZ, USA; SynCardia), experimental second-generation continuous flow devices (Cleveland Clinic Continuous-Flow Total Artificial Heart (CFTAH), Cleveland Clinic, OH, USA; BiVACOR, Huntington Beach, CA, USA), and hybrid bioprosthetic designs (Aeson®, Carmat SA, Paris, FRA) [11,1,17]. Representative contemporary TAH platforms are shown in Figure 1.

The SynCardia TAH received United States Food and Drug Administration (FDA) approval on October 15, 2004, and remains the only TAH with U.S. FDA approval for bridge-to-transplantation [18]. In its pivotal 10-year clinical trial, 79 of 81 implanted patients were successfully bridged to heart transplantation, compared to 46 of 81 control patients who did not receive the implant [18]. Bridged patients who underwent transplantation had one-year and five-year survival rates of 86% and 64%, respectively [18]. Registry data indicate that over 1,800 SynCardia implants have been performed worldwide, though precise implant counts vary by reporting source [11].

The Aeson® bioprosthetic TAH received European Conformity (CE) marking in 2020. This device incorporates bovine pericardial tissue as blood-contacting surfaces, glutaraldehyde-treated hybrid membranes, and an autoregulated electro-hydraulic actuation system that varies beat rate (30 to 150 beats per minute (bpm)) and stroke volume (maximum 65 mL) based on venous return [1,17]. Computational fluid dynamics fluid-structure interaction modeling reported in the Aeson® investigational studies demonstrates shear stress below 10 dynes/cm² in 90% of the flow volume, with peak values reaching 1500 dynes/cm² in less than 0.001% of blood volume [1,17,2]. Key structural and engineering differences among contemporary TAH platforms are summarized in Table 2.

**Table 2:** Comparison of major TAH devices TAH: Total artificial heart, CE: European conformity, vWF: von Willebrand factor

Device	Approval status	Flow type	Blood-contacting surface	Key features	Reported outcomes
SynCardia	FDA approval (2004), CE mark	Pulsatile pneumatic	Polyurethane, mechanical valves	70 mL or 50 mL ventricles, external driver	79% bridged to transplant in pivotal trial [18]
Aeson®	CE mark (2020)	Pulsatile, autoregulated	Bovine pericardial tissue, bioprosthetic valves	Embedded sensors, autoregulated mode, 30-150 bpm	No vWF multimer loss in 10-patient series [2,19]
BiVACOR	Investigational	Continuous flow (rotary)	Titanium, magnetically levitated rotor	Single moving part, biventricular support	Preclinical/early feasibility studies

Intended Clinical Role

The TAH implantation is indicated for patients with biventricular failure who require immediate ventricular replacement due to imminent mortality risk. Clinical indications include bridge to transplantation (most common), bridge to decision in unstable patients requiring hemodynamic stabilization before transplant eligibility determination, and, in limited investigational contexts, destination therapy [13]. The TAH therapy differs fundamentally from left ventricular assist device (LVAD) therapy. The LVADs augment native left ventricular (LV) function while preserving the right ventricle and native cardiac structures. The TAHs completely remove both ventricles and all four cardiac valves, eliminating all endogenous cardiac activity [11,17]. This total excision renders recipients entirely dependent on the mechanical apparatus for survival, with no potential for native recovery.

The Persistent Problem

Despite 50 years of engineering refinement, available data indicate that total artificial hearts have not demonstrated survival outcomes comparable to heart transplantation [18,13]. Single-center data from the University Hospital of Rennes in France (83 LVAD, 20 TAH implantations as of March 2022) indicate that 36.6% of LVAD and 55% of TAH patients proceeded to transplantation [20]. In this single-center series (n=20 TAH recipients), stroke occurred in 40% of TAH patients; however, these findings should be interpreted within the limitations of small sample size and center-specific practice patterns [20]. Significant bleeding events complicated 38% of LVAD and 50% of TAH courses [20] in the same single-center report.

The FDA approval data from 2004 reported 70% one-year survival among bridged patients after transplant, representing combined device and transplant survival rather than destination therapy outcomes alone [18]. According to the International Society for Heart and Lung Transplantation (ISHLT) registry, adult heart transplantation achieves 85% to 90% one-year survival and approximately 75% five-year survival among recipients surviving the first post-transplant year [21]. Data from the Interagency Registry for Mechanically Assisted Circulatory Support (INTERMACS) further characterize outcomes following TAH implantation. The central question is not whether TAHs have improved, but why complete mechanical replacement of cardiac function remains biologically incomplete after five decades of engineering effort, substantial federal investment, and clinical trials. Direct survival comparisons between TAH recipients, LVAD recipients, and transplant populations must be interpreted cautiously, given differences in baseline acuity, comorbid burden, and selection criteria.

Recurrent failure domains

Hemocompatibility Failure

Non-physiologic flow field and shear stress: Mechanical circulatory support systems expose blood to supraphysiologic shear forces that differ fundamentally from natural cardiovascular hemodynamics. Normal human circulatory wall shear stress ranges from 14-36 dynes/cm² in large arteries and 20-72 dynes/cm² in arterioles [1]. Pathologic shear stress associated with stenotic vessels and prothrombotic conditions ranges from 36-450 dynes/cm² [1]. First-generation positive displacement TAHs (SynCardia, Jarvik 7) generate peak shear stress of 100-500 dynes/cm², approximately 27 times the physiologic maximum [1,3]. By comparison, shear in rotary continuous flow LVADs is substantially higher: axial flow pumps (HeartMate II (Toratec, Viersen, DEU) and Jarvik 2000) produce shear of 2,000-24,000 dynes/cm², while centrifugal flow pumps generate 500-3,200 dynes/cm² [1,4-8]. SynCardia TAHs contain four mechanical valves (two inlet, two outlet) that create additional shear zones and flow separation areas [1].

Acquired von Willebrand Syndrome: High shear conditions induce conformational unfolding of von Willebrand factor (vWF) multimers, exposing the A2 domain to cleavage by ADAMTS13 (a disintegrin and metalloprotease with thrombospondin type-1 motif 13). Loss of high molecular weight multimers (>10,000 kDa) produces acquired von Willebrand syndrome biochemically identical to congenital type 2A disease [1]. This defect has been reported in the majority of LVAD recipients, with some studies documenting its occurrence within hours of device implantation [1]. The Aeson® bioprosthetic TAH, with its tissue interfaces and reduced shear profile, does not induce acquired von Willebrand syndrome. A study of the first 10 Aeson® recipients with 2,087 cumulative support days detected no change in vWF multimer profiles, in contrast to HeartMate II and HeartMate III control patients [2,19]. A negative correlation was observed between cardiac output, beat rate, and high molecular weight multimers, indicating vWF functions as a biological sensor of blood flow and pulsatility [2]. This exception demonstrates that improved hemocompatibility is achievable, though no TAH has yet integrated complete physiologic autoregulation, durable tissue interfaces, and driver autonomy in a fully approved implant.

Subclinical erythrocyte trauma and hemolysis: Overt hemolysis, measured by plasma-free hemoglobin, is not consistently present in modern TAH patients. In the Aeson® cohort, no correlation was found between plasma-free hemoglobin and cardiac output or beat rate over 2,087 days [2]. However, subclinical erythrocyte damage detectable through flow cytometry, microparticle formation, and impaired erythrocyte deformability occurs throughout mechanical circulatory support [1]. Cell-free hemoglobin has been shown in experimental studies to scavenge nitric oxide approximately 1,000 times more efficiently than erythrocyte-encapsulated hemoglobin, potentially inducing local microvascular vasoconstriction, platelet activation, and endothelial dysfunction [1]. Red cell microparticles expose phosphatidylserine, which can serve as catalytic platforms for tenase and prothrombinase complex assembly [1].

The hemocompatibility paradox: The TAHs promote both thrombotic and bleeding diatheses, a paradox explicable by blood-material incompatibility rather than pump action alone. Systemic anticoagulation (target international normalized ratio 2.0-3.0 for SynCardia plus antiplatelet therapy) remains necessary, indicating that mechanical valves and polyurethane surfaces are thrombogenic [1,13]. However, anticoagulated patients with acquired von Willebrand syndrome develop mucosal bleeding, gastrointestinal arteriovenous malformation bleeding, and intracranial hemorrhage [1]. Dose adjustment cannot resolve this paradox. The disorder is device-induced rather than a disease state. Five decades of mechanical optimization have reduced but not eliminated hemocompatibility failure because blood has evolved to contact vascular endothelium, not polymer, metal, or fixed xenogeneic tissue [1].

Infection and Interface Failure

**Figure 2:**
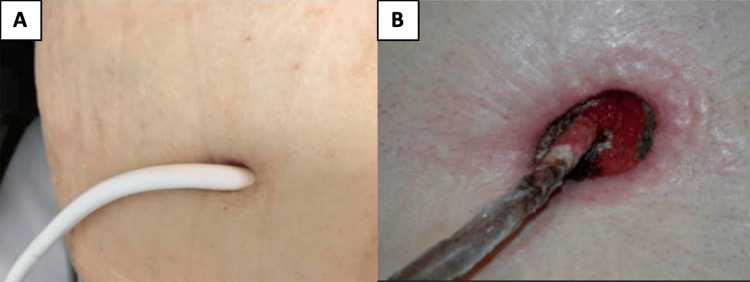
Representative driveline exit-site infection A: Uninfected driveline exit site; B: Driveline exit-site infection demonstrating tissue destruction and granulation tissue Image adapted from *Ventricular Assist Device-Specific Infections* by Qu et al., 2021, J Clin Med, 10(3):453 [22]. Distributed under the Creative Commons Attribution 4.0 License.

Driveline vulnerability as a permanent portal: All currently marketed TAH platforms rely on a permanent percutaneous interface for external power and/or drive support. This transcutaneous conduit breaches the integumentary barrier, establishing permanent communication between the non-sterile external environment and the sterile mediastinal space. Representative clinical pathology of a driveline exit-site infection is shown in Figure 2 [22].

In the Rennes series, 21% of LVAD recipients developed driveline infections [20]. Scanning electron microscopy and micro-CT of explanted infected drivelines revealed microbial biofilms proliferating along the velour surface, with inadequate host tissue integration creating microgaps between biomaterial and adjacent tissue [9]. In all nine drivelines examined, organisms isolated at various depths along the velour segment correlated with clinical exit site swab cultures [9]. Biofilms formed microcolonies within the driveline tunnel using human tissue as a substrate, not merely adhering to device surfaces [9]. Biofilm migration occurred through dispersal seeding, rendering these infections resistant to antimicrobial therapy regardless of antibiotic susceptibility profiles [9].

Historical antecedents: A Brno research center performed 66 long-term TAH implant experiments in calves and identified infection and sepsis as the leading causes of death [10]. Investigators noted that biomaterial infection represented a novel clinical phenomenon fundamentally different from conventional infections: "Such infections are chronic; they are resistant to host defense mechanisms as well as antibiotic treatment since the nature of these microorganisms has adapted as a result of their protection by the biofilm of certain types of bacteria on the surfaces of the implant biomaterials" [10]. This 2003 description accurately predicted findings later validated in human explant studies [9]. Researchers observed diminished immune protection during infectious courses in TAH recipients, high antimicrobial resistance, and a frequent combination of infection with thrombosis [10]. Multifactorial prevention and therapy improved survival in their series from 39 to 293 days but did not eliminate infection [10].

Device-specific considerations: The Aeson® TAH, utilizing tissue interfaces without mechanical valves, eliminates some bacterial seeding sites. However, it retains a percutaneous driveline (smaller caliber than pneumatic drivers) and remains susceptible to ascending infection. No commercially approved, fully implantable TAH using transcutaneous energy transfer has been achieved, despite decades of investigation [11]. Available evidence indicates that chronic percutaneous interfaces remain associated with persistent infection risk [9,10].

Physiologic Mismatch

Loss of autoregulation: The native heart modulates cardiac output on a beat-to-beat basis through intrinsic mechanisms (Starling's law, force-frequency relationship) and extrinsic neurohormonal regulation (sympathetic and parasympathetic innervation, circulating catecholamines, and atrial natriuretic peptide). Total artificial hearts completely remove this regulatory apparatus.

SynCardia TAH patients depend on external pneumatic drivers that maintain constant beat rate and fill volumes, with output changes occurring passively based on venous inflow within ventricular fill rate limitations [1]. This represents preload responsiveness rather than autoregulation. The Aeson® TAH incorporates pressure sensors that regulate pump flow based on target inflow pressures (range 5-20 mmHg), enabling an autoregulated mode that restores circadian hemodynamic fluctuations and preload-responsive output changes [1,17]. This constitutes significant engineering advancement toward physiologic integration. Neither device restores neurohormonal feedback. Ventricular mechanoreceptor baroreceptors and their afferent signals are absent. Atrial excision or exclusion abolishes atrial natriuretic peptide secretion, normally released in response to atrial stretch [11]. The renin-angiotensin-aldosterone system responds to renal perfusion pressure but without cardiac-derived control.

Non-physiologic flow profiles: The SynCardia TAH produces pulsatile flow with pressure waveforms resembling native physiology but with constant systolic ejection periods and non-compliant ventricles [1]. Continuous flow TAHs (Cleveland Clinic CFTAH, BiVACOR) generate non-pulsatile or minimally pulsatile flow. Acute animal experiments with the Cleveland Clinic CFTAH demonstrated that induced pulmonary arterial pulsation of 18 mmHg and systemic arterial pulse pressure of 18 mmHg could be achieved through sinusoidal modulation of speed waveforms, substantially lower than native pulse pressure [23].

The microcirculatory effects of chronic non-pulsatile flow remain incompletely characterized. Long-term LVAD recipients exhibit impaired microvascular reactivity, reduced functional capillary density, and diminished flow-mediated dilation [1]. Renal autoregulation, dependent on myogenic response and tubuloglomerular feedback, functions suboptimally under non-pulsatile conditions despite compensatory mechanisms [11]. Pump function replacement does not constitute cardiac physiology replacement. The heart functions not only as a pressure-volume generator but also as a sensory organ, endocrine organ, and integral component of neurohumoral regulation. Complete mechanical excision removes these functions, and engineering has not yet recapitulated them.

Immune and Inflammatory Activation

Contrasting evidence: Inflammatory responses to TAH implantation differ across device platforms. Comprehensive immunophenotyping of nine Aeson® recipients at 12-month follow-up showed no significant modulation of leukocyte counts, no inflammatory cytokine profile differences compared to pre-implantation conditions (all p > 0.05), and no significant modulation of circulating immune cell subpopulations by multiplex cluster of differentiation expression patterns [19]. This absence of peripheral blood inflammatory indicators over 12 months after Aeson® implantation contrasts sharply with historical TAH experience and LVAD literature. Researchers concluded that bioprosthesis surface modification effectively reduced chronic foreign body response [14]. This finding requires contextual interpretation. The Aeson® cohort is limited (n=9) with a 12-month follow-up, and peripheral blood cytokine profiles may not fully capture localized mediastinal inflammation. The device retains a percutaneous driveline, and chronic low-grade inflammation at the exit site remains possible without systemic cytokine elevation.

Historical TAH experience documented substantial inflammatory activation. The Brno animal series observed diminished immune defense in TAH recipients during infectious progression [4]. Immunothrombosis, characterized by combined infectious complications and thrombus formation, was frequently observed [4]. Complete replacement may impose a greater biomaterial burden than LV assist devices due to a larger implanted surface area and more extensive surgical dissection [5,4]. While hemocompatible tissue interfaces may suppress systemic inflammatory responses, long-term data remain limited [12].

Patient Selection and Systems Limitations

Challenge in diagnosing right ventricular (RV) failure: Determining irreversible biventricular failure, particularly distinguishing irreversible RV failure from secondary right ventricular failure due to LV failure, remains fundamental to appropriate TAH patient selection [13]. Existing predictive scores and algorithms demonstrate variable accuracy, reflecting an incomplete understanding of RV pathophysiology [13]. Some LVAD recipients develop RV failure after implantation, which may prove permanent [13]. Some centers now employ temporary LV assistance before TAH implantation to unmask RV failure in borderline cases [13].

Identified risk factors: Risk factor analysis has revealed counterintuitive findings. Smoking history constitutes the strongest independent risk factor for mortality following TAH implantation [13,24]. Prior cardiac surgery also independently predicts mortality; patients with prior sternotomy undergoing TAH have worse outcomes than those receiving primary implantation [13-16]. Age over 55 years is not strictly contraindicated but prompts careful consideration, particularly in bridge-to-transplant populations facing potentially long waiting periods [13]. Relative contraindications include severe malnutrition, an elevated frailty index, thrombophilia (an absolute contraindication for mechanical valve TAHs, potentially addressable with bioprosthetic valves), and severe psychosocial limitations such as body dysmorphic disorders impairing device management [13].

Anatomic constraints: Historically, the typical TAH recipient has been male with a body surface area exceeding 1.8 m² [13]. Device size may complicate implantation in women or smaller patients. Introduction of smaller artificial ventricles (50 mL SynCardia version with fixed cardiac output of 3.8-5.1 L/min versus 70 mL version with cardiac output of 6.3-7.5 L/min) has expanded eligibility but introduces flow limitations potentially inadequate for some patients with high-output requirements, such as sepsis [1,13]. Thoracic anterior-posterior distance measurement and three-dimensional reconstruction using virtual device fit now constitute standard preoperative planning with CT-guided virtual device fit [13,25].

The selection paradox: As TAH outcomes improve, the patient population referred for implantation becomes increasingly complex, i.e., more INTERMACS profiles, greater comorbidity burden, and more frequent previous cardiac surgery. In the Rennes experience, 30% of implanted patients were INTERMACS profile 1 (critical cardiogenic shock), representing sicker patients than those in published literature, with correspondingly worse long-term survival [20]. This referral bias obscures actual device effectiveness and creates an apparent survival ceiling potentially attributable to patient selection rather than device performance. Current evidence suggests that optimized patient selection may improve survival, though selection cannot fully resolve fundamental biologic incompatibility [13,20,14-16].

What these failure patterns suggest

The Heart's Functions Beyond Pumping

Fifty years of accumulated experience suggest the need for reconceptualization as the human heart is more than solely a pump. It is an endocrine organ: atrial myocytes produce and release atrial natriuretic peptide in response to stretch, regulating sodium excretion, vascular tone, and intravascular volume. Ventricular myocytes produce B-type natriuretic peptide. The TAH implantation ablates these humoral signals, eliminating a key component of cardiorenal integration [11].

As an immunologic organ, the heart contains resident macrophages, mast cells, and lymphocytes participating in innate and adaptive immunity. Cardiac transplantation transfers donor immune cells to the recipient; TAH implantation simply removes them [11,12]. The heart is a neurohumoral sensor: afferent baroreflex input, essential for blood pressure regulation, originates from vascular mechanoreceptors. Their absence compromises reflex responses to hemodynamic perturbation [11,1].

It is a trophic organ where cardiac tissue secretes growth factors such as neuregulin 1, supporting cardiomyocyte survival and vascular maintenance. Long-term effects of complete heart excision on systemic trophic support remain uncharacterized [11]. Available evidence indicates that pump function equivalence alone does not address the multisystem nature of heart failure, which manifests not only through insufficient flow but also through congestion, neurohormonal activation, inflammation, and end-organ dysfunction [11,1,13].

Blood-Material Interaction, the Central Limiting Factor

Fifty years of intensive engineering effort, from initial Jarvik implants to magnetically levitated continuous-flow rotors to bioprosthetic tissue interfaces, have not fully solved hemocompatibility. It is important to acknowledge that contemporary surface bioengineering has substantially advanced hemocompatibility science. Strategies such as heparin-bonded polymers, nitric oxide-modulating surfaces, and biomaterial interface optimization have demonstrated measurable reductions in platelet activation and inflammatory signaling in both experimental and clinical settings [2,19,12]. These advances illustrate that thrombogenicity is modifiable rather than fixed. Nevertheless, no currently approved TAH fully reproduces the dynamic, living endothelial interface capable of continuous repair, adaptive signaling, and systemic vascular integration.

The Aeson® exemplifies this progress, with preservation of von Willebrand multimers, absence of measurable hemolysis, and no detectable systemic inflammatory activation in early cohorts [2,19]. However, clinical experience remains limited, the device continues to rely on a percutaneous driveline, and, in the U.S., it is currently available only within an FDA-approved feasibility study. Available evidence indicates that even advanced biologic interfaces have not eliminated the broader constraints imposed by chronic blood-material interaction.

Blood evolved over 500 million years to interact with vascular endothelium, a living, antithrombotic, immunologically active cellular monolayer that regulates coagulation, inflammation, and vascular tone. Any artificial surface, however sophisticated, represents a deviation from this biologic standard. Cardiopulmonary bypass exhibits similar hemocompatibility defects, such as acquired von Willebrand syndrome, platelet dysfunction, and complement activation, but remains tolerable due to limited exposure of hours rather than years. The TAH recipients require indefinite exposure. The temporal accumulation of hemocompatibility-related adverse events may reflect intrinsic biologic constraints that have not yet been fully overcome by current engineering approaches.

Biologic Replacement Through Transplantation

Cardiac transplantation achieves superior outcomes not through better pump function. Artificial hearts generate equivalent cardiac output, pressure, and flow. Transplantation's advantage is biologic. Transplantation replaces the failed organ with living tissue: vascularized, eventually innervated, and capable of autoregulation, endocrine secretion, and neurohumoral integration. The graft endothelium is endogenous and releases nitric oxide, prostacyclin, and ectonucleotidases that actively inhibit thrombosis. The graft myocardium contains functional baroreceptors and releases natriuretic peptides.

Immunosuppression carries significant costs, including calcineurin inhibitors, antiproliferative agents, and corticosteroids with attendant risks of infection, malignancy, nephrotoxicity, and metabolic derangement. However, registry data demonstrate that long-term survival is achievable with transplantation [21]. The relevant comparison is not between device complications and immunosuppression risks but between biologic integration and mechanical replacement.

This is not an argument against developing TAHs. It is an argument for evidence-based expectations regarding what TAHs can and cannot accomplish. Current data indicate they can restore cardiac output and extend survival in carefully selected patients as a bridge to transplantation. Long-term durability comparable to transplantation has not been demonstrated.

Clinical implications

The findings of this review carry several implications for current clinical practice and patient counseling. First, patient selection remains paramount. Available evidence suggests optimal TAH candidates are those with irreversible biventricular failure who are candidates for subsequent transplantation [13-16]. Patients with prior sternotomy, smoking history, or significant comorbidities face elevated risk and require particularly careful evaluation [13,24]. Second, expectations for destination therapy require calibration. While TAHs can restore hemodynamics, registry data indicate that long-term outcomes comparable to transplantation have not yet been achieved [18,20,21]. Patients and families should understand that adverse event rates remain substantial, particularly for stroke and bleeding [20] (Table 3). Third, the choice between LVAD and TAH should consider not only hemodynamic requirements but also the implications of native cardiac tissue preservation. For patients without irreversible right ventricular failure, LVAD therapy preserves endocrine, immunologic, and neurohumoral functions that may contribute to long-term stability [11,1]. Fourth, infection prevention strategies require ongoing optimization. Despite advances in driveline design and surgical technique, percutaneous interfaces remain associated with infectious risk that can persist indefinitely [9,10]. Finally, centers offering TAH therapy should participate in registries to contribute to the evidence base regarding optimal patient selection and long-term outcomes [6,16].

Future directions

Partial Support Strategies

The LVAD experience provides instructive lessons. The field evolved from first-generation pulsatile devices (HeartMate XVE, Novacor (Saint-Antoine, FRA)) with one-to-two-year durability limitations to continuous-flow second-generation devices (HeartMate II) with 80% two-year survival to third-generation magnetically levitated centrifugal pumps (HeartMate 3) with five-year survival exceeding 50% and substantial improvement in stroke-free survival [11,1]. Preservation of native cardiac architecture may contribute to improved long-term outcomes with LVADs compared to TAHs. The recipient's right ventricle remains in place. Native aortic and mitral valves continue functioning. Pericardial excision is not performed. Even dysfunctional native myocardial tissue continues secreting natriuretic peptides, providing baroreceptor afferent signaling and maintaining an active endothelial surface. The LVADs augment native function; TAHs replace it. This distinction carries profound implications. Partial support strategies should remain the dominant paradigm for mechanical circulatory support. Total replacement should be reserved for patients with no alternative, such as biventricular failure without recovery potential, massive ventricular thrombus, ventricular septal rupture, or complex congenital heart disease ineligible for transplantation [11,13].

Biologic-Mechanical Hybrid Concepts

The Aeson® demonstrates that hybrid devices (combining synthetic structural components with biologic blood-contacting surfaces) can achieve superior hemocompatibility compared to all-synthetic devices [2,19,12]. This principle warrants extension. Potential directions include endothelialization of blood-contacting surfaces through pre-implantation cell seeding or in situ capture of circulating endothelial progenitor cells [11]. Tissue-engineered heart muscle patches embedded into artificial ventricles could provide contractile augmentation and autoregulation. Decellularized whole-heart scaffolds recellularized with patient-derived induced pluripotent stem cells might offer anatomically correct, immunologically compatible grafts [11]. These approaches remain investigational, facing substantial challenges in scalability, sterility, durability, and regulatory approval. However, they acknowledge the fundamental lesson of the past half-century: synthetic materials alone cannot achieve indefinite biologic compatibility.

Smart Physiologic Control Systems

The Aeson®'s autoregulated mode (using embedded pressure sensors to adjust beat rate and stroke volume based on venous return) represents significant progress in physiologic responsiveness [1,17,2]. Further refinement remains possible. Future systems could incorporate additional sensors (oxygen saturation, hemoglobin concentration, activity level) and machine learning algorithms to predict hemodynamic demands and proactively adjust device parameters. Closed-loop control systems integrating device output with physiologic native signals (heart rate variability, respiratory sinus arrhythmia) might achieve partial neurohumoral integration. However, no control algorithm can restore endocrine function or baroreceptor afferent signaling. Smart systems can optimize pump output, but cannot replace the heart as a sensory organ.

Should Total Replacement Be Abandoned?

Given that TAHs have not yet demonstrated destination therapy durability comparable to transplantation, an important question for the field is whether greater emphasis should be placed on partial support and regenerative strategies. Total artificial hearts have demonstrated unequivocal efficacy as bridge-to-transplantation devices. The 79% transplant rate in the SynCardia pivotal trial and cumulative successfully bridged patients represent genuine achievements [11,18,26]. For patients dying of biventricular failure with no other options, TAHs save lives that would otherwise be lost. The case for destination therapy TAH is less compelling. No device has received U.S. FDA approval for this indication [18]. Registry data suggest survival outcomes remain below those achieved with transplantation and current LVAD therapy [11,18,20,16,27]. The adverse event burden reported in some series, a 40% stroke rate and 50% bleeding rate, is substantial for elective permanent replacement (Table 3) [20].

**Table 3:** Adverse event rates in selected TAH series TAH: Total artificial heart, INTERMACS: Interagency Registry for Mechanically Assisted Circulatory Support, LVAD: Left ventricular assist device

Series	Device	N	Stroke rate	Bleeding rate	Infection rate	Transplant rate
Rennes single-center [20]	LVAD (mixed)	83	23.5%	38%	21% (driveline)	36.6%
Rennes single-center [20]	TAH (mixed)	20	40%	50%	Not specified separately	55%
INTERMACS registry [16]	TAH	450	11% (at six months)	22% (at six months)	15% (at six months)	58% (at six months)

Resource and ethical considerations are substantial. National inpatient cost analyses demonstrate that expenditures related to advanced mechanical circulatory support and transplant procedures are substantial, with annual LVAD-related hospital costs approaching hundreds of millions of U.S. dollars and frequently exceeding costs associated with heart transplantation, reflecting the high economic burden of advanced mechanical support in contemporary practice [28,29]. In an era of limited healthcare resources, the economic implications of destination therapy TAH warrant careful evaluation relative to continued investment in LVAD optimization and regenerative approaches. However, raw expenditure alone does not define value. Formal cost-effectiveness analyses in durable mechanical circulatory support have demonstrated that cost per quality-adjusted life year (QALY) is highly sensitive to survival duration and complication burden. In destination therapy LVAD populations, cost per QALY has improved over successive device generations as stroke, pump thrombosis, and rehospitalization rates declined, underscoring the importance of event-free survival in determining economic value [30]. Comparable survival-adjusted economic modeling for TAH destination therapy remains limited, and complication-driven costs may substantially influence long-term value assessment.

Fully artificial hearts have faced persistent challenges not solely due to engineering limitations but also because the human heart functions as a biologically integrated organ embedded within coagulation, immune, endocrine, and neurohumoral systems. Mechanical replication of flow is not equivalent to physiologic replacement. The field must acknowledge this limitation and realign its goals accordingly. Total artificial hearts are likely to remain superior bridge-to-transplantation devices for patients with biventricular failure. Long-term equivalence to transplantation has not been demonstrated. Fifty years of evidence support this conclusion.

This editorial is limited by reliance on published registry data, selected single-center experiences, and mechanistic studies rather than systematic meta-analysis. Reporting heterogeneity, evolving device generations, and differences in patient selection criteria may influence interpretation of outcome comparisons. Conclusions should therefore be understood within the context of these methodological limitations.

Fifty years of development have defined the capabilities and limitations of TAHs. They provide reliable circulatory restoration in patients with otherwise fatal biventricular failure and have enabled thousands of individuals to survive until transplantation. In this defined role, they represent genuine therapeutic achievement. Cumulative evidence suggests that complete mechanical heart replacement is subject to intrinsic biologic limitations. Chronic blood exposure to synthetic or fixed biologic surfaces maintains thrombogenicity, hemorrhage risk, and inflammatory potential despite progressive design improvements. Percutaneous energy transmission perpetuates infectious vulnerability. Most fundamentally, excision of native cardiac tissue eliminates endocrine, immunologic, endothelial, and neurohumoral functions that pressure sensors and control algorithms cannot recreate. Transplantation succeeds not by generating superior flow but by restoring living tissue capable of autoregulation and systemic physiologic integration. This contrast illuminates the central lesson of TAH experience: the heart is not solely a hydraulic pump but a biologically embedded organ. Future progress will likely require hybrid biologic-mechanical strategies, improved surface endothelialization, and smarter control systems. However, expectations must align with biologic reality. Total artificial hearts are effective bridge devices. As destination therapy, current evidence suggests they may be constrained by biologic as well as technical limitations. Recognition of these limitations is essential for rational allocation of research effort and clinical application.
